# Sample size determination and power analysis using the G*Power software

**DOI:** 10.3352/jeehp.2021.18.17

**Published:** 2021-07-30

**Authors:** Hyun Kang

**Affiliations:** Department of Anesthesiology and Pain Medicine, Chung-Ang University College of Medicine, Seoul, Korea; Hallym University, Korea

**Keywords:** Biometry, Correlation of data, Research personnel, Sample size, Software

## Abstract

Appropriate sample size calculation and power analysis have become major issues in research and publication processes. However, the complexity and difficulty of calculating sample size and power require broad statistical knowledge, there is a shortage of personnel with programming skills, and commercial programs are often too expensive to use in practice. The review article aimed to explain the basic concepts of sample size calculation and power analysis; the process of sample estimation; and how to calculate sample size using G*Power software (latest ver. 3.1.9.7; Heinrich-Heine-Universität Düsseldorf, Düsseldorf, Germany) with 5 statistical examples. The null and alternative hypothesis, effect size, power, alpha, type I error, and type II error should be described when calculating the sample size or power. G*Power is recommended for sample size and power calculations for various statistical methods (F, t, χ^2^, Z, and exact tests), because it is easy to use and free. The process of sample estimation consists of establishing research goals and hypotheses, choosing appropriate statistical tests, choosing one of 5 possible power analysis methods, inputting the required variables for analysis, and selecting the “calculate” button. The G*Power software supports sample size and power calculation for various statistical methods (F, t, χ^2^, z, and exact tests). This software is helpful for researchers to estimate the sample size and to conduct power analysis.

## Introduction

### Background/rationale

If research can be conducted among the entire population of interest, the researchers would obtain more accurate findings. However, in most cases, conducting a study of the entire population is impractical, if not impossible, and would be inefficient. In some cases, it is more accurate to conduct a study of appropriately selected samples than to conduct a study of the entire population. Therefore, researchers use various methods to select samples representing the entire population, to analyze the data from the selected samples, and to estimate the parameters of the entire population, making it very important to determine the appropriate sample size to answer the research question [1,2]. However, the sample size is often arbitrarily chosen or reflects limits of resource allocation. However, this method of determination is often not scientific, logical, economical, or even ethical.

From a scientific viewpoint, research should provide an accurate estimate of the therapeutic effect, which may lead to evidence-based decisions or judgments. Studies with inappropriate sample sizes or powers do not provide accurate estimates and therefore report inappropriate information on the treatment effect, making evidence-based decisions or judgments difficult. If the sample size is too small, even if a large therapeutic effect is observed, the possibility that it could be caused by random variations cannot be excluded. In contrast, if the sample size is too large, too many variables—beyond those that researchers want to evaluate in the study—may become statistically significant. Some variables may show a statistically significant difference, even if the difference is not meaningful. Thus, it may be difficult to determine which variables are valid.

From an economic point of view, studies with too large a sample size may lead to a waste of time, money, effort, and resources, especially if availability is limited. Studies with too small a sample size provide low power or imprecise estimates; therefore, they cannot answer the research questions, which also leads to a waste of time, money, effort, and resources. For this reason, considering limited resources and budget, sample size calculation and power analysis may require a trade-off between cost-effectiveness and power [3,4].

From an ethical point of view, studies with too large a sample size cause the research subjects to waste their effort and time, and may also expose the research subjects to more risks and inconveniences.

Considering these scientific, economic, and ethical aspects, sample size calculation is critical for research to have adequate power to show clinically meaningful differences. Some investigators believe that underpowered research is unethical, except for small trials of interventions for rare diseases and early phase trials in the development of drugs or devices [5].

Although there have been debates on sample size calculation and power analysis [3,4,6], the need for an appropriate sample size calculation has become a major trend in research [1,2,7-11].

### Objectives

This study aimed to explain the basic concepts of sample size calculation and power analysis; the process of sample estimation; and how to calculate sample size using the G*Power software (latest ver. 3.1.9.7; Heinrich-Heine-Universität Düsseldorf, Düsseldorf, Germany; http://www.gpower.hhu.de/) with 5 statistical examples.

## Basic concept: what to know before performing sample size calculation and power analysis

In general, the sample size calculation and power analysis are determined by the following factors: effect size, power (1-β), significance level (α), and type of statistical analysis [1,7]. The International Committee of Medical Journal Editors recommends that authors describe statistical methods with sufficient detail to enable a knowledgeable reader with access to the original data to verify the reported results [12], and the same principle should be followed for the description of sample size calculation or power analysis. Thus, the following factors should be described when calculating the sample size or power.

### Null and alternative hypotheses

A hypothesis is a testable statement of what researchers predict will be the outcome of a trial. There are 2 basic types of hypotheses: the null hypothesis and the alternative hypothesis. H0: The null hypothesis is a statement that there is no difference between groups in terms of a mean or proportion. H1: The alternative hypothesis is contradictory to the null hypothesis.

### Effect size

The effect size shows the difference or strength of relationships. It also represents a minimal clinically meaningful difference [12]. As the size, distribution, and units of the effect size vary between studies, standardization of the effect size is usually performed for sample size calculation and power analysis.

The choice of the effect size may vary depending on the study design, outcome measurement method, and statistical method used. Of the many different suggested effect sizes, the G*Power software automatically provides the conventional effect size values suggested by Cohen by moving the cursor onto the blank region of “effect size” in the “input parameters” field [12].

### Power, alpha, type I error, and type II error

A type I error, or false positive, is the error of rejecting a null hypothesis when it is true, and a type II error, or false negative, is the error of accepting a null hypothesis when the alternative hypothesis is true.

Intuitively, type I errors occur when a statistically significant difference is observed, despite there being no difference in reality, and type II errors occur when a statistically significant difference is not observed, even when there is truly a difference (Table 1). In Table 1, the significance level (α) represents the maximum allowable limit of type I error, and the power represents the minimum allowable limit of accepting the alternative hypothesis when the alternative hypothesis is true.

If the results of a statistical analysis are non-significant, there are 2 possibilities for the non-significant results: (1) correctly accepting the null hypothesis when the null hypothesis is true and (2) erroneously accepting the null hypothesis when the alternative hypothesis is true. The latter occurs when the research method does not have enough power. If the power of the study is not known, it is not possible to interpret whether the negative results are due to possibility (1) or possibility (2). Thus, it is important to consider power when planning studies.

## Process of sample size calculation and power analysis

In many cases, the process of sample size calculation and power analysis is too complex and difficult for common programs to be feasible. To calculate sample size or perform power analysis, some programs require a broad knowledge of statistics and/or software programming, and other commercial programs are too expensive to use in practice.

To avoid the need for extensive knowledge of statistics and software programming, herein, we demonstrate the process of sample size and power calculation using the G*Power software, which has a graphical user interface (GUI). The G*Power software is easy to use for calculating sample size and power for various statistical methods (F, t, χ^2^, Z, and exact tests), and can be downloaded for free at www.psycho.uni-duesseldorf.de/abteilungen/aap/gpower3. G*Power also provides effect size calculators and graphics options. Sample size and power calculations using G*Power are generally performed in the following order.

### First, establish the research goals and hypotheses

The research goals and hypotheses should be elucidated. The null and alternative hypotheses should be presented, as discussed above.

### Second, choose appropriate statistical tests

G*Power software provides statistical methods in these 2 ways.

#### Distribution-based approach

Investigators can select the distribution-based approach (exact, F, t, χ^2^, and z tests) using the “test family” drop-down menu.

#### Design-based approach

Investigators can select the design-based approach using the “statistical test” drop-down menu. This can also be carried out by selecting the variable (correlation and regression, means, proportions, variance, and generic) and the study design for which statistical tests are performed from the test menu located at the top of the screen and sub-menu.

### Third, choose 1 of 5 possible power analysis methods

This choice can be made by considering the variables to be calculated and the given variables. Researchers can select 1 of the 5 following types in the “type of power analysis” drop-down menu (Table 2).

An a priori analysis is a sample size calculation performed before conducting the study and before the design and planning stage of the study; thus, it is used to calculate the sample size N, which is necessary to determine the effect size, desired α level, and power level (1-β). As an a priori analysis provides a method for controlling type I and II errors to prove the hypothesis, it is an ideal method of sample size and power calculation.

In contrast, a post-hoc analysis is typically conducted after the completion of the study. As the sample size N is given, the power level (1-β) is calculated using the given N, the effect size, and the desired α level. Post-hoc power analysis is a less ideal type of sample size and power calculation than a priori analysis as it only controls α, and not β. Post-hoc power analysis is criticized because the type II error calculated using the results of negative clinical trials is always high, which sometimes leads to incorrect conclusions regarding power [13,14]. Thus, post-hoc power analysis should be cautiously used for the critical evaluation of studies with large type II errors.

### Fourth, input the required variables for analysis and select the “calculate” button

In the “input parameters” area of the main window of G*Power, the required variables for analysis can be entered. If information is available to calculate the effect size from a pilot study or a previous study, the effect size calculator window can be opened by checking the “determine→” button, and the effect size can be calculated using this information.

## Five statistical examples of using G*Power

**Fig. 1.**
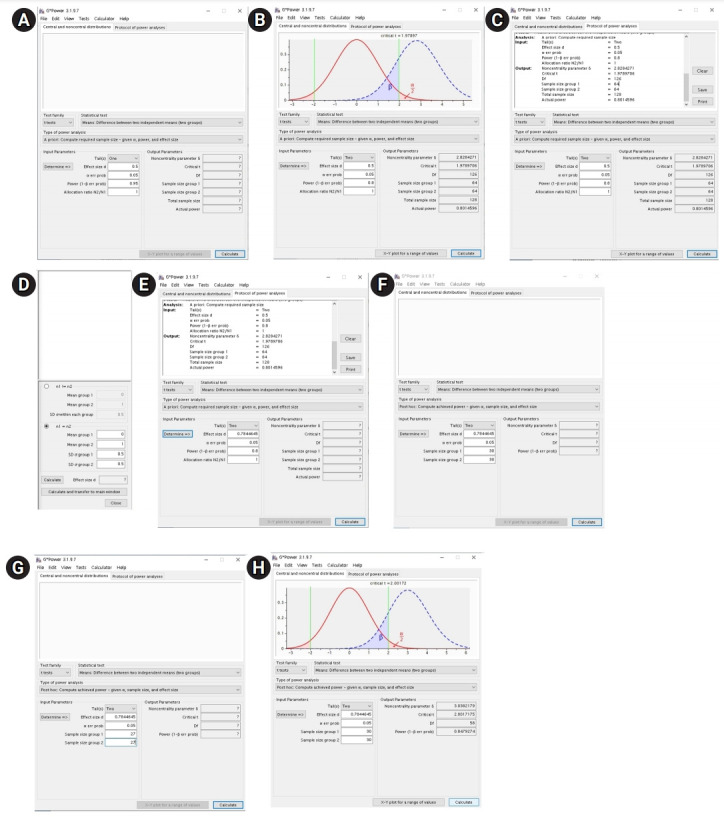
Using the G*Power software for the 2-independent sample t-test. (A) Main window before a priori sample size calculation using an effect size, (B) main window after a priori sample size calculation using an effect size, (C) main window showing the protocol for power analyses, (D) effect size calculator window, (E) plot window, (F) main window before a priori sample size calculation not using an effect size, (G) main window after a priori sample size calculation not using an effect size, and (H) main window before post-hoc power analysis.

G*Power shows the following menu bars at the top of the main window when the program starts up: “file,” “edit,” “view,” “tests,” “calculator,” and “help” (Fig. 1A). Under these menu bars, there is another row of tabs, namely, “central and noncentral distributions” and “protocol of power analyses.” The “central and noncentral distribution” tab shows the distribution plot of null and alternative hypotheses and α and β values (Fig. 1B). Moreover, checking the “protocol of power analyses” tab shows the results of the calculation, including the name of the test, type of power analysis, and input and output parameters, which can be cleared, saved, and printed using the “clear,” “save,” and “print” buttons, respectively, which are located at the right side of the results (Fig. 1C).

In the middle of the main screen, drop-down menus named “test family,” “statistical test,” and “type of power analysis” are located, where the appropriate statistical test and type of power analysis can be selected.

In the lower part, in the “input parameters” field, information regarding the sample size calculation or power analysis can be entered, and in the “output parameters” field, the results for sample size calculation or power analysis will appear. On the left side of the “input parameters” field, there is a “determine→” button. Clicking the “determine→” button will lead to the effect size calculator window, where the effect size can be calculated by inputting information (Fig. 1D).

In the lowest part of the main screen, there are 2 buttons named “X-Y plot for a range of values” and “calculate.” Checking the “X-Y plot for a range of values” button leads to the plot window, where the graphs or tables for the α error probability, power (1-β error probability), effect size, or total sample size can be obtained (Fig. 1E). Checking the “calculate” button enables the calculation of the sample size or power.

### Examples 1. Two-sample or independent t-test: t-test

The 2-sample t-test (also known as the independent t-test or Student t-test) is a statistical test that compares the mean values of 2 independent samples. The null hypothesis is that the difference in group means is 0, and the alternative hypothesis is that the difference in group means is different from 0.

H_0_: μ_1_-μ_2_=0
H_1_: μ_1_-μ_2_≠0
H_0_: null hypothesis
H_1_: alternative hypothesis
μ_1_, μ_2_: means of each sample

#### Example: a priori

Let us assume that researchers are planning a study to investigate the analgesic efficacy of 2 drugs. Drug A has traditionally been used for postoperative pain control and drug B is a newly developed drug. In order to compare the efficacy of drug B with that of drug A, a pain score using a Visual Analog Scale (VAS) will be measured at 6 hours postoperation. The researchers want to determine the sample size for the null hypothesis to be rejected with a 2-tailed test, α=0.05, and β=0.2. The number of patients in each group is equal.

**When the effect size is determined:** If the effect size to be found is determined, the procedure for calculating the sample size is very easy. For the sample size calculation of a statistical test, G*Power provides the effect size conventions as “small,” “medium” and “large,” based on Cohen’s suggestions [12]. These provide conventional effect size values that are different for different tests.

Moving the cursor onto the blank of “effect size” in the “input parameters” field will show the conventional effect size values suggested by G*Power. For the sample size calculation of the t-test, G*Power software provides the conventional effect size values of 0.2, 0.5, and 0.8 for small, medium, and large effect sizes, respectively. In this case, we attempted to calculate the sample size using a medium effect size (0.5).

After opening G*Power, go to “test>means>two independent groups.” Then, the main window shown in Fig. 1A appears.

Here, as the sample size is calculated before the conduction of the study, set “type of power analysis” as “A priori: compute required sample size-given α, power, and effect size.” Since the researchers decided to use a medium effect size, 2-sided testing, α=0.05, β=0.2, and an equal sample size in both groups, select “two” for the “tail(s)” drop-down menu and input 0.5 for the blank of “effect size d,” 0.05 for the blank of “α err prob,” 0.8 for the blank of “power (1-β err prob),” and 1 for the blank of “Allocation ratio N2/N1.” Upon pushing the “calculate” button, the sample size for group 1, the sample size for group 2, and the total sample size will be computed as 64, 64, and 128, respectively, as shown in the output parameters of the main window (Fig. 1B).

Upon clicking on the “protocol of power analyses” tab in the upper part of the window, the input and output of the power calculation can be automatically obtained. The results can be cleared, saved, and printed using the “clear,” “save,” and “print” buttons, respectively (Fig. 1C).

**When the effect size is not determined:** Next, let us consider the case in which the effect size is not determined in the study design similar to that described above. In fact, the effect size is not determined on many occasions and it is debatable whether a determined effect size can be applied when designing a study. Thus, when the effect size value is arbitrarily determined, we should provide a logical rationale for the arbitrarily selected effect size. When the effect size is not determined, the effect size value can only be assumed using the variables from other previous studies, pilot studies, or experiences.

In this example, as there was no previous study comparing the efficacy of drug A to that of drug B, we will assume that the researchers conducted a pilot study with 10 patients in each group, and the means and standard deviations (SDs) of the VAS for drug A and B were 7±3 and 5±2, respectively.

In the main window, push the “determine” button; then, the effect size calculator window will appear (Fig. 1D). Here, there are 2 options: “n1!=n2” and “n1=n2.” Because we assume an equal sample size for 2 groups, we check the “n1=n2” box.

Since the pilot study showed that the mean and SD of the VAS for drug A and drug B were 7±3 and 5±2, respectively, input 7 for “mean group 1,” 3 for “SD σ group 1,” 5 for “mean group 2,” and 2 for “SD σ group 2,” and then click the “calculate and transfer to main window” button.

Then, the corresponding effect size “0.7844645” will be automatically calculated and appear in the blank space for “effect size d” in the main window (Fig. 1F).

As described above, we decided to use 2-sided testing, α=0.05, β=0.2, and an equal sample size in both groups. We selected “two” in the “tail(s)” drop-down menu and entered 0.05 for “α err prob,” 0.8 for “power (1-β err prob),” and 1 for “allocation ratio N2/N1.” Clicking the “calculate” button computes “sample size group 1,” “sample size group 2,” and “total sample size” as 27, 27, and 54, respectively, in the output parameters area (Fig. 1G).

#### Example: post hoc

A clinical trial comparing the efficacy of 2 analgesics, drugs A and B, was conducted. In this study, 30 patients were enrolled in each group, and the means and SDs of the VAS for drugs A and B were 7±3 and 5±2, respectively. The researchers wanted to determine the power using a 2-tailed test, α=0.05, and β=0.2.

After opening G*Power, go to “test>means>two independent groups.” In the main window, select “type of power analysis” as “post hoc: compute achieved power-given α, sample size and effect size,” and then push the “determine” button. In the effect size calculator window, since a clinical trial was conducted with equal group sizes, showing that the means and SDs of the VAS for drug A and B were 7±3 and 5±2, respectively, check the “n1=n2” box and input 7 for “mean group 1,” 3 for “SD σ group 1,” 5 for “mean group 2,” and 2 for “SD σ group 2,” and then click the “calculate and transfer to main window” button. Then, the corresponding effect size “0.7844645” will be calculated automatically and appear in the blank space for “effect size d” in the main window (Fig. 1H). Because we decided to use 2-sided testing, α=0.05, and an equal sample size (n=30) in both groups, we selected “two” in the tail(s) drop-down menu, and entered 0.05 for “α err prob,” 30 for “sample size group 1,” and 30 for “sample size group 2.” Pushing the “calculate” button will compute “power (1-β err prob)” as 0.8479274.

### Examples 2. Dependent t-test: t-test

The dependent t-test (paired t-test) is a statistical test that compares the means of 2 dependent samples. The null hypothesis is that the difference between the means of dependent groups is 0, and the alternative hypothesis is that the difference between the means of dependent groups is not equal to 0.

H_0_: μ_1_-μ_2_=0
H_1_: μ_1_-μ_2_≠0
H_0_: null hypothesis
H_1_: alternative hypothesis
μ_1_, μ_2_: means of each sample from dependent groups

#### Example: a priori

Imagine a study examining the effect of a diet program on weight loss. In this study, the researchers plan to enroll participants, weigh them, enroll them in a diet program, and weigh them again. The researchers want to determine the sample size for the null hypothesis to be rejected with a 2-tailed test, α=0.05, and β=0.2.

**When the effect size is determined:** After opening G*Power, go to “test>means>two dependent groups (matched pairs).”

**Fig. 2.**
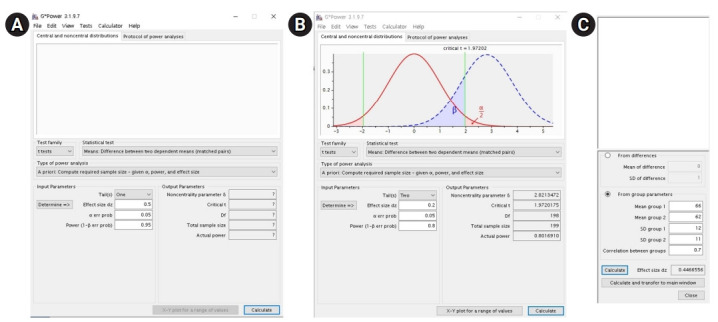
Using the G*Power software for the dependent t-test. (A) Main window before a priori sample size calculation using an effect size, (B) main window after a priori sample size calculation using an effect size, and (C) effect size calculator window.

In the main window, set “type of power analysis” as “a priori: compute required sample size-given α, power, and effect size” (Fig. 2A). Since the researchers decided to use 2-sided testing, α=0.05, and β=0.2, select “two” for the tail(s) drop-down menu, and input 0.05 for the blank of “α err prob” and 0.8 for the blank of “power (1-β err prob).” Unlike the case for the t-test, as the number of participants is expected to be equal in the paired t-test, the blank for “allocation ratio N2/N1” is not provided. In this case, calculate the sample size using a small effect size (0.2); thus, input 0.2 for “effect size dz.” Upon pushing the “calculate” button, the total sample size will be computed as 199 in the output parameter area (Fig. 2B).

**When the effect size is not determined:** Suppose the researchers conducted a pilot study to calculate the sample size for the study investigating the effect of the diet program on weight loss. In this pilot study, the mean and SD before and after the diet program were 66±12 kg and 62±11 kg, respectively, and the correlation between the weights before and after the diet program was 0.7.

Click the “determine” button in the main window and then check the “from group parameters” box from the 2 options “from differences” and “from group parameters” in the effect size calculator window, as the group parameters are known.

Since the pilot study showed that the means and SDs of the results before and after the diet program were 66±12 kg and 62±11 kg, respectively, and the correlation between the results before and after the diet program was 0.7, input 66 for “mean group 1,” 62 for “mean group 2,” 12 for “SD group 1,” 11 for “SD group 2,” and 0.7 for “correlation between groups,” and then click the “calculate and transfer to main window” button (Fig. 2C). Then, the corresponding effect size “0.4466556” will be calculated automatically and appear at the blank space for “effect size dz” in the main screen and in the effect size calculator screen. In the main window, as we decided to use 2-sided testing, α=0.05, and β=0.2, we will select “two” in the “tail(s)” drop-down menu, and input 0.05 for “α err prob” and 0.8 for “power (1-β err prob).” Pushing the “calculate” button will compute the “total sample size” as 42.

#### Example: post hoc

Assume that a clinical trial comparing participants’ weight before and after the diet program was conducted. In this study, 100 patients were enrolled, the means and SDs before and after the diet program were 66±12 kg and 62±11 kg, respectively, and the correlation between the weights before and after the diet program was 0.7. The researchers wanted to determine the power for 2-tailed testing and α=0.05.

After opening G*Power, go to “test>means>two dependent groups (matched pairs).” In the main screen, select “type of power analysis” as “post hoc: compute achieved power-given α, sample size and effect size,” and then push the “determine” button. In the effect size calculator screen, select the “from group parameters” check box from the 2 options “from differences” and “from group parameters” as the group parameters are known. In this effect size calculator window, as a clinical trial showed that the means and SDs of the results before and after the diet program were 66±12 kg and 62±11 kg, respectively, and the correlation between the results before and after the diet program was 0.7, input 66 for “mean group 1,” 62 for “mean group 2,” 12 for “SD group 1,” 1 for “SD group 2,” and 0.7 for “correlation between groups,” and then click the “calculate and transfer to main window” button. Then, the corresponding effect size “0.4466556” will be calculated automatically and appear in the blank space for “effect size dz” in the main window and in the effect size calculator window.

In the main window, as we decided to use 2-sided testing and α=0.05 and the enrolled number of patients was 100, select “two” in the “tail(s)” drop-down menu and input 0.05 for “α err prob” and 100 for “total sample size.” Pushing the “calculate” button will compute “power (1-β err prob)” as 0.9931086.

### Examples 3. One-way analysis of variance: F-test

One-way analysis of variance (ANOVA) is a statistical test that compares the means of 3 or more samples. The null hypothesis is that all k means are identical, and the alternative hypothesis is that at least 2 of the k means differ.

H_0_: μ_1_=μ_2_= ⋯=μ_k_
H_1_: the means are not all equal
H_0_: null hypothesis
H_1_: alternative hypothesis
μ_1_, μ_2_, ⋯ μ_k_: means of each sample from the independent groups (1, 2, ⋯, k)

The assumptions of the ANOVA test are as follows: (1) independence of observations, (2) normal distribution of dependent variables, and (3) homogeneity of variance.

#### Example: a priori

Assume that a study investigated the effects of 4 analgesics: A, B, C, and D. Pain will be measured at 6 hours postoperatively using a VAS. The researchers wanted to determine the sample size for the null hypothesis to be rejected at α=0.05 and β=0.2.

**When the effect size is determined:** For the ANOVA test, Cohen suggested the effect sizes of “small,” “medium,” and “large” as 0.1, 0.25, and 0.4, respectively [12], and G*Power provides conventional effect size values when the cursor is moved onto the “effect size” in the “input parameters” field. In this case, we calculated the sample size using a medium effect size (0.25).

After opening G*Power, go to “test>means>many groups: ANOVA: one-way (one independent variable).” In the main window, set “type of power analysis” as “a priori: compute required sample size-given α, power, and effect size.” Since we decided to use a medium effect size, α=0.05, and β=0.2, we enter 0.25 for “effect size f,” 0.05 for “α err prob,” and 0.8 for “power (1-β err prob).” Moreover, as we compared 4 analgesics, we input 4 for “number of groups.” Pushing the “calculate” button computes the total sample size as 180, as shown in the “total sample size” in the “output parameters” area.

**When the effect size is not determined:** Assume that a pilot study showed the following means and SDs (of VAS) at 6 hours postoperation for drugs A, B, C, and D: 2±2, 4±1, 5±1, and 5±2, respectively, in 5 patients for each group.

Push the “determine” button and the effect size calculator window will appear. Here, we can find 2 options: “effect size from means” and “effect size from variance.” Select “effect size from means” in the “select procedure” drop-down menu and select 4 in the “number of groups” drop-down menu.

Here, as G*Power does not provide the common SD, we must calculate this. The formula for the common SD is as follows:

$$s_{\mathrm{pooled}}=\sqrt{\frac{(n_{1}-1){S_{1}}^{2}+(n_{2}-1){S_{2}}^{2}+(n_{3}-1){S_{3}}^{2}+(n_{4}-1){S_{4}}^{2}}{\left((n_{1}-1)+(n_{2}-1)+(n_{3}-1)+(n_{4}-1)-4\right)}}$$

S_pooled_: common SD

S_1_, S_2_, S_3_, S_4_: SDs in each group

n_1_, n_2_, n_3_, n_4_: numbers of patients in each group

Using the above formula, we obtain the following:

$$S_{\mathrm{pooled}}=\sqrt{\frac{(5-1)2^{2}+(5-1)1^{2}+(5-1)1^{2}+(5-1)2^{2}}{\left((5-1)+(5-1)+(5-1)+(5-1)-4\right)}}$$

Herein, the common SD will be 1.58. Thus, input 1.58 into the blank of “SD σ within each group.” As the means and numbers of patients in each group are 2, 4, 5, and 5 and 5, 5, 5, and 5, respectively, input each value, and then click the “calculate and transfer to main window” button. Then, the corresponding effect size “0.7751550” will be automatically calculated and appear in the blank for “effect size f” in the main window.

Since we decided to use 4 groups, α=0.05, and β=0.2, input 4 for “number of groups,” 0.05 for “α err prob,” and 0.8 for “power (1-β err prob).” Pushing the “calculate” button computes the total sample size as 24, as shown in the “total sample size” in the “output parameters” area.

#### Example: post hoc

Assume that a clinical trial showed the following means and SDs of VAS at 6 hours postoperation in drugs A, B, C, and D : 2±2, 4±1, 5±1, and 5±2, respectively, in 20 patients for each group. The researchers want to determine the power with 2-tailed testing, α=0.05, and β=0.2.

After opening G*Power, go to “test>means>many groups: ANOVA: one-way (one independent variable).” In the main screen, select “type of power analysis” as “post hoc: compute achieved power-given α, sample size and effect size,” and then push the “determine” button to show the effect size calculator screen.

Using the above formula, the common SD is 1.58. Thus, input 1.58 into the blank of “SD σ within each group.” As the means and numbers of patients in each group are 2, 4, 5, and 5, and 20, 20, 20, and 20, respectively, input these values into the corresponding blank; next, click the “calculate and transfer to main window” button.

Then, the corresponding effect size “0.7751550” and the total number of patients will be calculated automatically and appear in the blanks for “effect size f” and “total sample size” on the main screen.

Since the clinical trial used 4 groups and we decided to use α=0.05, input 4 for “number of groups” and 0.05 for “α err prob.” Pushing the “calculate” button will compute the power as 0.9999856 at “power (1-β err prob)” in the “output parameters” area.

### Examples 4. Correlation–Pearson r

A correlation is a statistic that measures the relationship between 2 continuous variables. The Pearson correlation coefficient is a statistic that shows the strength of the relationship between 2 continuous variables. The Greek letter ρ (rho) represents the Pearson correlation coefficient in a population, and r represents the Pearson correlation coefficient in a sample. The null hypothesis is that the Pearson correlation coefficient in the population is 0, and the alternative hypothesis is that the Pearson correlation coefficient in the population is not equal to 0.

H_0_: ρ=0
H_1_: ρ≠0
H_0_: null hypothesis
H_1_: alternative hypothesis
ρ: Pearson correlation coefficient in the population

The Pearson correlation coefficient in the population (ρ, rho) ranges from −1 to 1, where −1 represents a perfect negative linear correlation, 0 represents no linear correlation, and 1 represents a perfect positive linear correlation. The coefficient of determination (ρ^2^) is calculated by squaring the Pearson correlation coefficient in the population (ρ) and is interpreted as “the percent of variation in one continuous variable explained by the other continuous variable” [15].

For correlations, Cohen suggested the effect sizes of “small,” “medium,” and “large” as 0.1, 0.3, and 0.5, respectively [12]. However, G*Power does not provide a sample size calculation using the effect size for correlations. Therefore, this article does not present a sample size calculation using the effect size. Instead, an example is provided of sample size calculation using the expected population Pearson correlation coefficient or coefficient of determination.

#### Example: a priori

Consider a hypothetical study investigating the correlation between height and weight in pediatric patients. The researchers wanted to determine the sample size for the null hypothesis to be rejected with 2-tailed testing, α=0.05, and β=0.2. In the pilot study, the Pearson correlation coefficient for the sample was 0.5.

After opening G*Power, go to “test>correlation and regression>correlation: bivariate normal model.” In the main screen, set “type of power analysis” as “a priori: compute required sample size-given α, power, and effect size.” Because we decided to use 2-sided testing, α=0.05, and β=0.2, the Pearson correlation coefficient in the null hypothesis is 0, and the Pearson correlation coefficient in the sample was 0.7, in the pilot study, select “two” for the “tail(s)” drop-down menu, and input 0.5 for “correlation ρ H1,” 0.05 for “α err prob,” 0.8 for “power (1-β err prob),” and 0 for “correlation ρ H1.” Upon pushing the “calculate” button, the total sample size will be computed as 29 in the “output parameters” area.

G*Power also provides an option to calculate the sample size using the coefficient of determination. If the coefficient of determination is known, push the “determine” button in the main window, input the value into the blank of “coefficient of determination ρ2,” and then click the “calculate and transfer to main window” button. Finally, push the “calculate” button to compute the total sample size.

#### Example: post hoc

Assume that a clinical trial investigated the correlation between height and weight in pediatric patients. In this study, 50 pediatric patients were enrolled, and the sample Pearson correlation coefficient was 0.5. The researchers wanted to determine the power at the 2-tailed and α=0.05 levels.

After opening G*Power, go to “test>correlation and regression>correlation: bivariate normal model.” In the main screen, select “type of power analysis” as “post hoc: compute achieved power-given α, sample size, and effect size.” Then, as the clinical trial showed that the sample Pearson correlation coefficient was 0.5 and the number of enrolled patients was 50, input 0.5 for “correlation ρ H1,” 0 for “correlation ρ H1,” and 50 for “total sample size.” As the researchers want to determine the power at the 2-tailed and α=0.05 levels, select “two” for the tail(s) drop-down menu and input 0.05 for “α err prob.”

Then, the corresponding power “0.9671566” will be calculated automatically and appear at the blank for “power (1-β err prob)” in the output parameter area of the main screen.

### Example 5. Two independent proportions: chi-square test

The chi-square test, also known as the χ^2^ test, is used to compare 2 proportions of independent samples. G*Power provides the options to compare 2 proportions of independent samples, namely “two independent groups: inequality, McNemar test,” “two independent groups: inequality, Fisher’s exact test,” “two independent groups: inequality, unconditional exact,” “two independent groups: inequality with offset, unconditional exact,” and “two independent groups: inequality, z-test.” In this article, we introduce the “two independent groups: inequality, z-test” because many statistical software programs provide similar options.

The null hypothesis is that the difference in proportions of independent groups is 0, and the alternative hypothesis is that the difference in proportions of independent groups is not equal to 0.

H_0_: π_1_-π_2_=0
H_1_: π_1_-π_2_≠0

As G*Power does not provide sample size calculation using the effect size for this option, this is not provided in this article; instead, an example of sample size calculation using the expected proportions of each group is provided.

#### Example: a priori

Assume that a study examined the effects of 2 treatments, for which the measure of the effect is a proportion. Treatment A has been traditionally used for the prevention of post-herpetic neuralgia, and treatment B is a newly developed treatment. The researchers wanted to determine the sample size for the null hypothesis to be rejected using 2-tailed testing, α=0.05, and β=0.2. The number of patients was equal in both groups. Suppose the researchers conducted a pilot study examining the effects of treatments A and B. In the pilot study, the proportions of post-herpetic neuralgia development were 0.3 and 0.1, respectively, for treatments A and B.

After opening G*Power, go to “test>proportions>two independent groups: inequality, z-test.” In the main window, set “type of power analysis” as “a priori: compute required sample size-given α, power, and effect size.”

Because we decided to use 2-sided testing, α=0.05, and β=0.2, there was an equal sample size in both groups, and the proportions of post-herpetic neuralgia development were 0.3 and 0.1 for treatments A and B, respectively, in the pilot study, select “two” for the “tail(s)” drop-down menu, and input 0.05 for “α err prob,” 0.8 for “power (1-β err prob),” 1 for “allocation ratio N2/N1,” and 0.3 and 0.1 for “proportion p2” and “proportion p1,” respectively.

Pushing the “calculate” button will compute “sample size group 1,” “sample size group 2,” and “total sample size” as 62, 62, and 124, respectively.

#### Example: post hoc

Assume that a clinical trial compared the effect of 2 treatments, A and B, on the incidence of post-herpetic neuralgia. In this study, researchers enrolled 101 and 98 patients, respectively, and post-herpetic neuralgia occurred in 31 and 9 patients after treatments A and B, respectively. The incidence of post-herpetic neuralgia in the groups that received treatments A and B was 0.307 (31/101) and 0.092 (9/98), respectively.

The researchers wanted to determine the power using 2-tailed testing and α=0.05. After opening G*Power, go to “test>proportions>two independent groups: inequality, z-test.” In the main screen, select “type of power analysis” as “post hoc: compute achieved power-given α, sample size and effect size.” Then, as a clinical trial showed that the incidence of post-herpetic neuralgia in the groups that received treatments A and B was 0.307 and 0.092, respectively, and the number of enrolled patients was 101 and 98, respectively, input 0.307 for “proportion p2,” 0.092 for “proportion p1,” 101 for “sample size group 2,” and 98 for “sample size group 1.”

As researchers wanted to determine the power for 2-tailed testing and α=0.05, select “two” for the tail(s) drop-down menu and input 0.05 for “α err prob.” Pushing the “calculate” button will compute “power (1-β err prob)” as 0.9715798 in the “output parameter” area of the main window.

## Sample size calculation considering the drop-out rate

When conducting a study, drop-out of study subjects or non-compliance to the study protocol is inevitable. Therefore, we should consider the drop-out rate when calculating the sample size. When calculating the sample size considering the drop-out rate, the formula for the sample size calculation is as below:

$N^{D}=\frac{N}{\left(1-d\right)}$
N: sample size before considering drop-out
d: expected drop-out rate
ND: sample size considering drop-out

Let us assume that a sample size for a study was calculated as 100. If the drop-out rate during the study process is expected to be 20% (0.2), the sample size considering drop-out will be 125.

$$\frac{100}{\left(1-0.2\right)}=125$$

## Conclusion

Appropriate sample size calculation and power analysis have become major issues in research and analysis. The G*Power software supports sample size and power calculation for various statistical methods (F, t, χ^2^, z, and exact tests). G*Power is easy to use because it has a GUI and is free. This article provides guidance on the application of G*Power to calculate sample size and power in the design, planning, and analysis stages of a study.

## Figures and Tables

**Table 1.** Types of statistical error and power and confidence levels

Null hypothesis	Decision	
	Accept H_0_	Reject H_o_
H_0_ is true	Correct (confidence level, 1-α)	Type I error (α)
H_0_ is false	Type II error (β)	Correct (power, 1-β)

H_0_, null hypothesis.

**Table 2.** Power analysis methods

Type	Independent variable	Dependent variable
1. A priori	Power (1-β), significance level (α), and effect size	N
2. Compromise	Effect size, N, q=β/α	Power (1-β), significance level (α)
3. Criterion	Power (1-β), effect size, N	Significance level (α), criterion
4. Post-hoc	Significance level (α), effect size, N	Power (1-β)
5. Sensitivity	Significance level (α), power (1-β), N	Effect size

N, sample size; q=β/α, error probability ratio, which indicates the relative proportionality or disproportionality of the 2 values.

## Supplementary materials

Supplement 1. Audio recording of the abstract.
